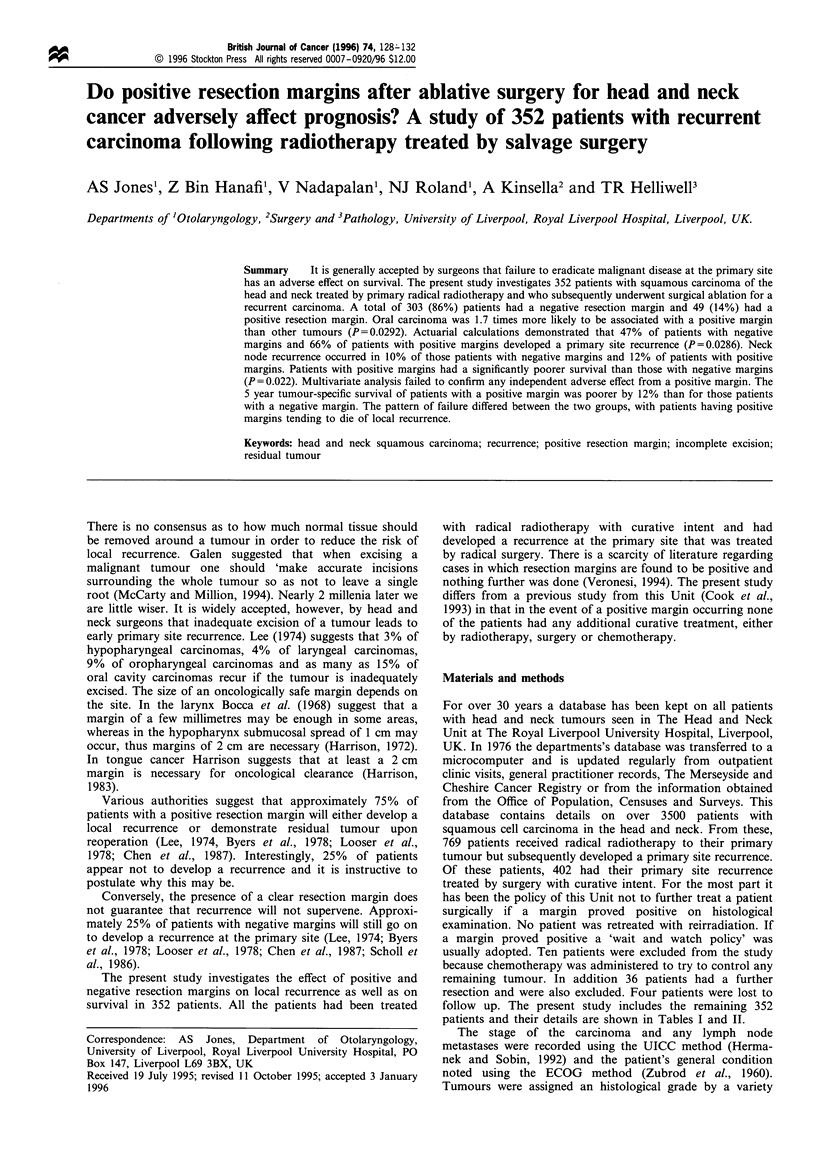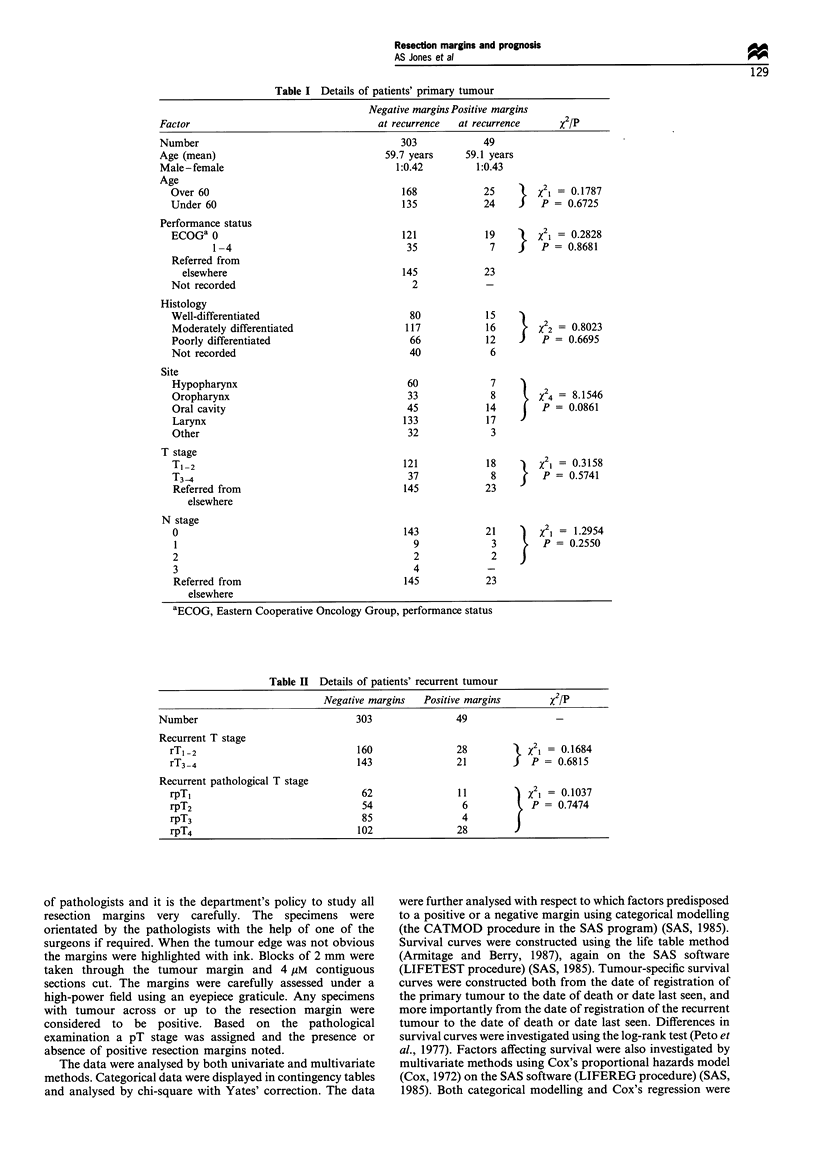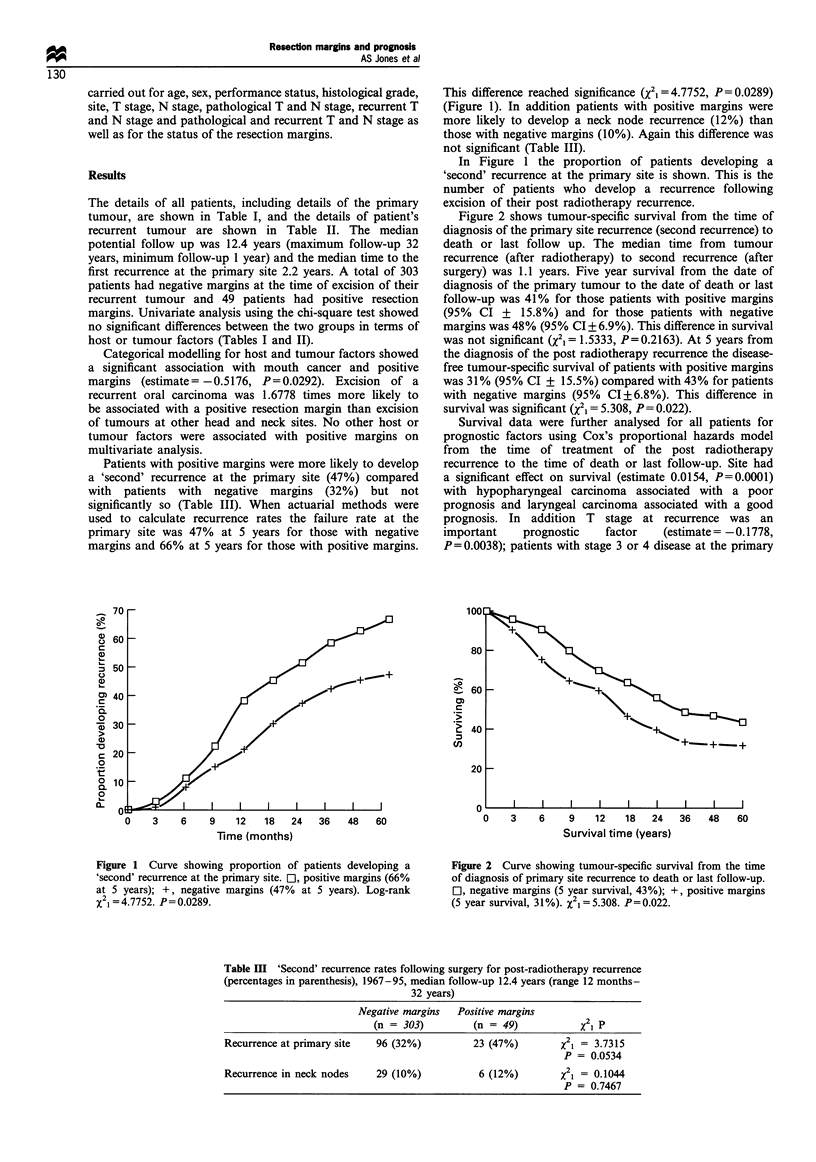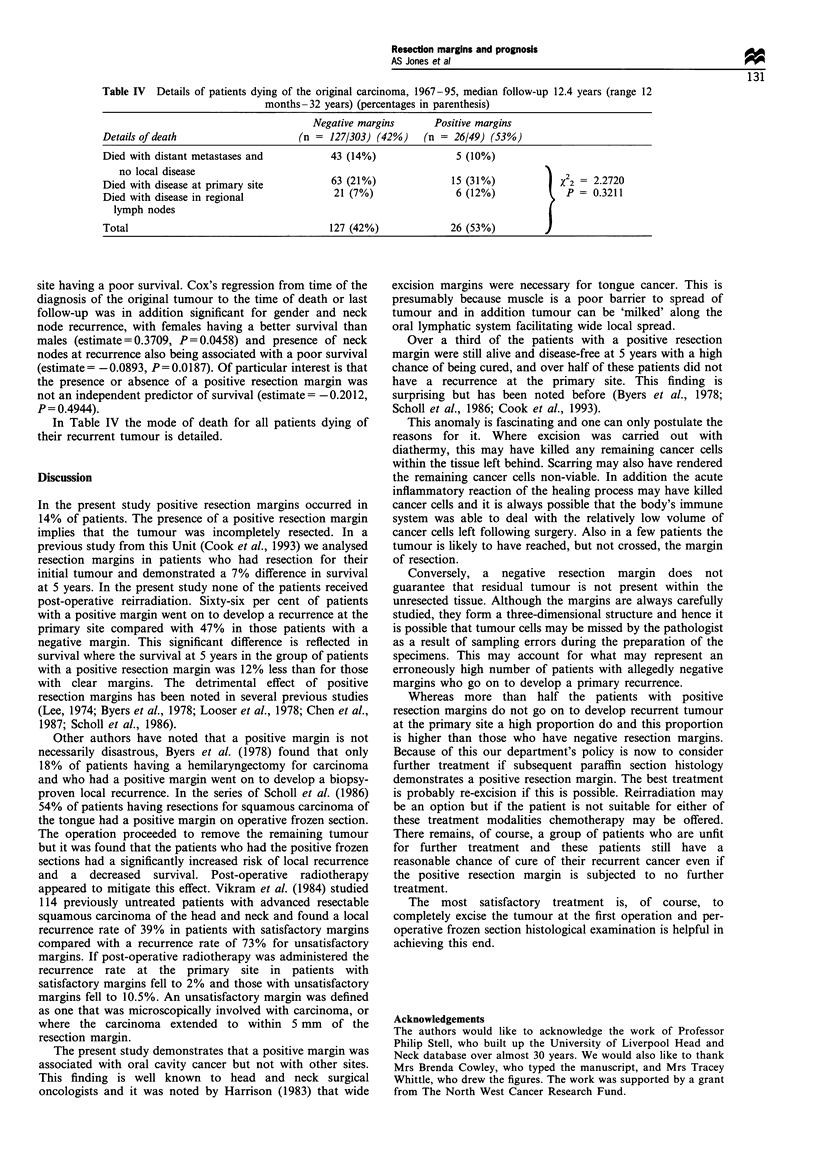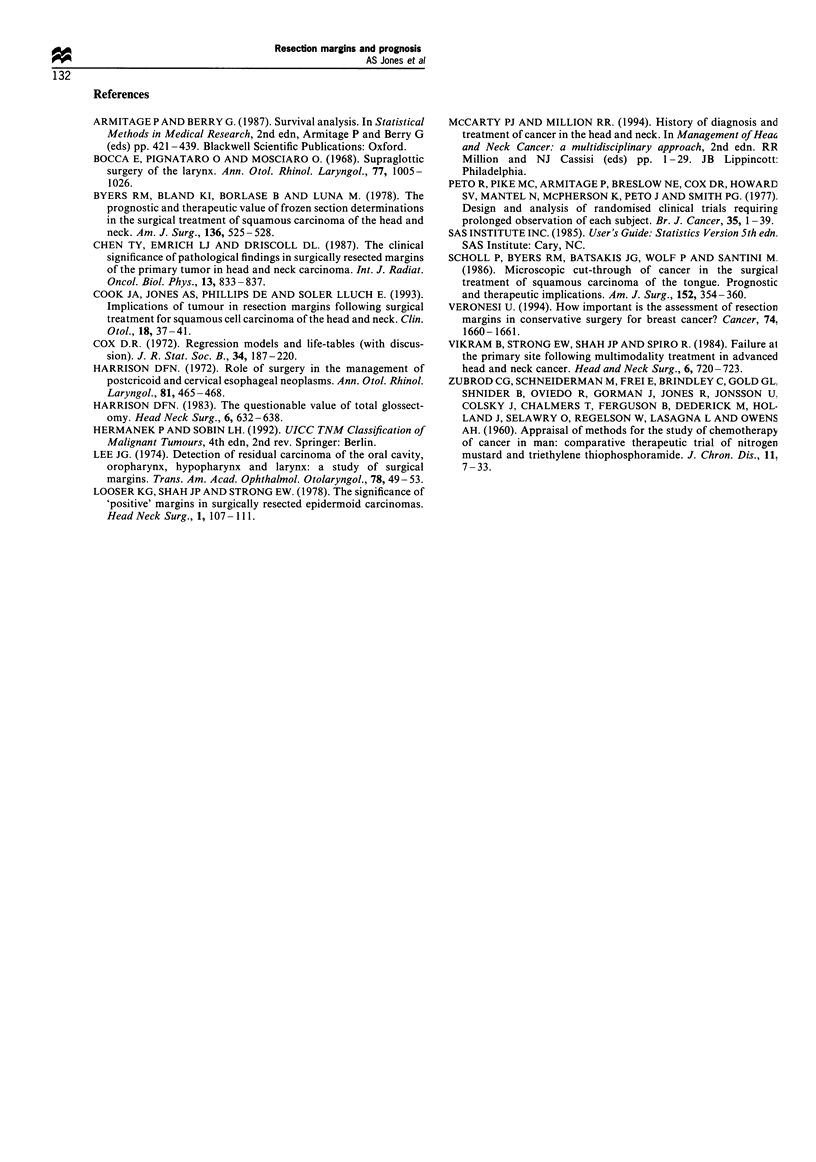# Do positive resection margins after ablative surgery for head and neck cancer adversely affect prognosis? A study of 352 patients with recurrent carcinoma following radiotherapy treated by salvage surgery.

**DOI:** 10.1038/bjc.1996.327

**Published:** 1996-07

**Authors:** A. S. Jones, Z. Bin Hanafi, V. Nadapalan, N. J. Roland, A. Kinsella, T. R. Helliwell

**Affiliations:** Department of Otolaryngology, University of Liverpool, Royal Liverpool Hospital, UK.

## Abstract

It is generally accepted by surgeons that failure to eradicate malignant disease at the primary site has an adverse effect on survival. The present study investigates 352 patients with squamous carcinoma of the head and neck treated by primary radical radiotherapy and who subsequently underwent surgical ablation for a recurrent carcinoma. A total of 303 (86%) patients had a negative resection margin and 49 (14%) had a positive resection margin. Oral carcinoma was 1.7 times more likely to be associated with a positive margin than other tumours (P = 0.0292). Actuarial calculations demonstrated that 47% of patients with negative margins and 66% of patients with positive margins developed a primary site recurrence (P = 0.0286). Neck node recurrence occurred in 10% of those patients with negative margins and 12% of patients with positive margins. Patients with positive margins had a significantly poorer survival than those with negative margins (P = 0.022). Multivariate analysis failed to confirm any independent adverse effect from a positive margin. The 5 year tumour-specific survival of patients with a positive margin was poorer by 12% than for those patients with a negative margin. The pattern of failure differed between the two groups, with patients having positive margins tending to die of local recurrence.


British Journal of Cancer (1996) 74, 128–132
© 1996 Stockton Press All rights reserved 0007-0920/96 $12.00

### Do positive resection margins after ablative surgery for head and neck cancer adversely affect prognosis? A study of 352 patients with recurrent carcinoma following radiotherapy treated by salvage surgery

AS Jones[1], Z Bin Hanafi[1], V Nadapalan[1], NJ Roland[1], A Kinsella[2] and TR Helliwell[3]

*Departments of [1]Otolaryngology, [2]Surgery and [3]Pathology, University of Liverpool, Royal Liverpool Hospital, Liverpool, UK.*

**Summary** It is generally accepted by surgeons that failure to eradicate malignant disease at the primary site has an adverse effect on survival. The present study investigates 352 patients with squamous carcinoma of the head and neck treated by primary radical radiotherapy and who subsequently underwent surgical ablation for a recurrent carcinoma. A total of 303 (86%) patients had a negative resection margin and 49 (14%) had a positive resection margin. Oral carcinoma was 1.7 times more likely to be associated with a positive margin than other tumours ($P=0.0292$). Actuarial calculations demonstrated that 47% of patients with negative margins and 66% of patients with positive margins developed a primary site recurrence ($P=0.0286$). Neck node recurrence occurred in 10% of those patients with negative margins and 12% of patients with positive margins. Patients with positive margins had a significantly poorer survival than those with negative margins ($P=0.022$). Multivariate analysis failed to confirm any independent adverse effect from a positive margin. The 5 year tumour-specific survival of patients with a positive margin was poorer by 12% than for those patients with a negative margin. The pattern of failure differed between the two groups, with patients having positive margins tending to die of local recurrence.

**Keywords:** head and neck squamous carcinoma; recurrence; positive resection margin; incomplete excision; residual tumour

There is no consensus as to how much normal tissue should be removed around a tumour in order to reduce the risk of local recurrence. Galen suggested that when excising a malignant tumour one should 'make accurate incisions surrounding the whole tumour so as not to leave a single root (McCarty and Million, 1994). Nearly 2 millenia later we are little wiser. It is widely accepted, however, by head and neck surgeons that inadequate excision of a tumour leads to early primary site recurrence. Lee (1974) suggests that 3% of hypopharyngeal carcinomas, 4% of laryngeal carcinomas, 9% of oropharyngeal carcinomas and as many as 15% of oral cavity carcinomas recur if the tumour is inadequately excised. The size of an oncologically safe margin depends on the site. In the larynx Bocca *et al.* (1968) suggest that a margin of a few millimetres may be enough in some areas, whereas in the hypopharynx submucosal spread of 1 cm may occur, thus margins of 2 cm are necessary (Harrison, 1972). In tongue cancer Harrison suggests that at least a 2 cm margin is necessary for oncological clearance (Harrison, 1983).

Various authorities suggest that approximately 75% of patients with a positive resection margin will either develop a local recurrence or demonstrate residual tumour upon reoperation (Lee, 1974, Byers *et al.*, 1978; Looser *et al.*, 1978; Chen *et al.*, 1987). Interestingly, 25% of patients appear not to develop a recurrence and it is instructive to postulate why this may be.

Conversely, the presence of a clear resection margin does not guarantee that recurrence will not supervene. Approximately 25% of patients with negative margins will still go on to develop a recurrence at the primary site (Lee, 1974; Byers *et al.*, 1978; Looser *et al.*, 1978; Chen *et al.*, 1987; Scholl *et al.*, 1986).

The present study investigates the effect of positive and negative resection margins on local recurrence as well as on survival in 352 patients. All the patients had been treated with radical radiotherapy with curative intent and had developed a recurrence at the primary site that was treated by radical surgery. There is a scarcity of literature regarding cases in which resection margins are found to be positive and nothing further was done (Veronesi, 1994). The present study differs from a previous study from this Unit (Cook *et al.*, 1993) in that in the event of a positive margin occurring none of the patients had any additional curative treatment, either by radiotherapy, surgery or chemotherapy.

### Materials and methods

For over 30 years a database has been kept on all patients with head and neck tumours seen in The Head and Neck Unit at The Royal Liverpool University Hospital, Liverpool, UK. In 1976 the departments's database was transferred to a microcomputer and is updated regularly from outpatient clinic visits, general practitioner records, The Merseyside and Cheshire Cancer Registry or from the information obtained from the Office of Population, Censuses and Surveys. This database contains details on over 3500 patients with squamous cell carcinoma in the head and neck. From these, 769 patients received radical radiotherapy to their primary tumour but subsequently developed a primary site recurrence. Of these patients, 402 had their primary site recurrence treated by surgery with curative intent. For the most part it has been the policy of this Unit not to further treat a patient surgically if a margin proved positive on histological examination. No patient was retreated with reirradiation. If a margin proved positive a 'wait and watch policy' was usually adopted. Ten patients were excluded from the study because chemotherapy was administered to try to control any remaining tumour. In addition 36 patients had a further resection and were also excluded. Four patients were lost to follow up. The present study includes the remaining 352 patients and their details are shown in Tables I and II.

The stage of the carcinoma and any lymph node metastases were recorded using the UICC method (Hermanek and Sobin, 1992) and the patient's general condition noted using the ECOG method (Zubrod *et al.*, 1960). Tumours were assigned an histological grade by a variety

Correspondence: AS Jones, Department of Otolaryngology, University of Liverpool, Royal Liverpool University Hospital, PO Box 147, Liverpool L69 3BX, UK
Received 19 July 1995; revised 11 October 1995; accepted 3 January 1996

**Table I** Details of patients' primary tumour

| *Factor* | *Negative margins at recurrence* | *Positive margins at recurrence* | $\chi^2$/P |
|---|---|---|---|
| Number | 303 | 49 | |
| Age (mean) | 59.7 years | 59.1 years | |
| Male–female | 1:0.42 | 1:0.43 | |
| Age | | | |
| Over 60 | 168 | 25 | $\chi^2_1 = 0.1787$ |
| Under 60 | 135 | 24 | $P = 0.6725$ |
| Performance status | | | |
| ECOG[a] 0 | 121 | 19 | $\chi^2_1 = 0.2828$ |
| 1–4 | 35 | 7 | $P = 0.8681$ |
| Referred from elsewhere | 145 | 23 | |
| Not recorded | 2 | – | |
| Histology | | | |
| Well-differentiated | 80 | 15 | |
| Moderately differentiated | 117 | 16 | $\chi^2_2 = 0.8023$ |
| Poorly differentiated | 66 | 12 | $P = 0.6695$ |
| Not recorded | 40 | 6 | |
| Site | | | |
| Hypopharynx | 60 | 7 | |
| Oropharynx | 33 | 8 | $\chi^2_4 = 8.1546$ |
| Oral cavity | 45 | 14 | $P = 0.0861$ |
| Larynx | 133 | 17 | |
| Other | 32 | 3 | |
| T stage | | | |
| $T_{1-2}$ | 121 | 18 | $\chi^2_1 = 0.3158$ |
| $T_{3-4}$ | 37 | 8 | $P = 0.5741$ |
| Referred from elsewhere | 145 | 23 | |
| N stage | | | |
| 0 | 143 | 21 | $\chi^2_1 = 1.2954$ |
| 1 | 9 | 3 | $P = 0.2550$ |
| 2 | 2 | 2 | |
| 3 | 4 | – | |
| Referred from elsewhere | 145 | 23 | |

[a]ECOG, Eastern Cooperative Oncology Group, performance status

**Table II** Details of patients' recurrent tumour

| | *Negative margins* | *Positive margins* | $\chi^2$/P |
|---|---|---|---|
| Number | 303 | 49 | – |
| Recurrent T stage | | | |
| $rT_{1-2}$ | 160 | 28 | $\chi^2_1 = 0.1684$ |
| $rT_{3-4}$ | 143 | 21 | $P = 0.6815$ |
| Recurrent pathological T stage | | | |
| $rpT_1$ | 62 | 11 | $\chi^2_1 = 0.1037$ |
| $rpT_2$ | 54 | 6 | $P = 0.7474$ |
| $rpT_3$ | 85 | 4 | |
| $rpT_4$ | 102 | 28 | |

of pathologists and it is the department's policy to study all resection margins very carefully. The specimens were orientated by the pathologists with the help of one of the surgeons if required. When the tumour edge was not obvious the margins were highlighted with ink. Blocks of 2 mm were taken through the tumour margin and 4 $\mu$M contiguous sections cut. The margins were carefully assessed under a high-power field using an eyepiece graticule. Any specimens with tumour across or up to the resection margin were considered to be positive. Based on the pathological examination a pT stage was assigned and the presence or absence of positive resection margins noted.

The data were analysed by both univariate and multivariate methods. Categorical data were displayed in contingency tables and analysed by chi-square with Yates' correction. The data were further analysed with respect to which factors predisposed to a positive or a negative margin using categorical modelling (the CATMOD procedure in the SAS program) (SAS, 1985). Survival curves were constructed using the life table method (Armitage and Berry, 1987), again on the SAS software (LIFETEST procedure) (SAS, 1985). Tumour-specific survival curves were constructed both from the date of registration of the primary tumour to the date of death or date last seen, and more importantly from the date of registration of the recurrent tumour to the date of death or date last seen. Differences in survival curves were investigated using the log-rank test (Peto *et al.*, 1977). Factors affecting survival were also investigated by multivariate methods using Cox's proportional hazards model (Cox, 1972) on the SAS software (LIFEREG procedure) (SAS, 1985). Both categorical modelling and Cox's regression were carried out for age, sex, performance status, histological grade, site, T stage, N stage, pathological T and N stage, recurrent T and N stage and pathological and recurrent T and N stage as well as for the status of the resection margins.

### Results

The details of all patients, including details of the primary tumour, are shown in Table I, and the details of patient's recurrent tumour are shown in Table II. The median potential follow up was 12.4 years (maximum follow-up 32 years, minimum follow-up 1 year) and the median time to the first recurrence at the primary site 2.2 years. A total of 303 patients had negative margins at the time of excision of their recurrent tumour and 49 patients had positive resection margins. Univariate analysis using the chi-square test showed no significant differences between the two groups in terms of host or tumour factors (Tables I and II).

Categorical modelling for host and tumour factors showed a significant association with mouth cancer and positive margins (estimate = −0.5176, $P = 0.0292$). Excision of a recurrent oral carcinoma was 1.6778 times more likely to be associated with a positive resection margin than excision of tumours at other head and neck sites. No other host or tumour factors were associated with positive margins on multivariate analysis.

Patients with positive margins were more likely to develop a 'second' recurrence at the primary site (47%) compared with patients with negative margins (32%) but not significantly so (Table III). When actuarial methods were used to calculate recurrence rates the failure rate at the primary site was 47% at 5 years for those with negative margins and 66% at 5 years for those with positive margins. This difference reached significance ($\chi^2_1 = 4.7752$, $P = 0.0289$) (Figure 1). In addition patients with positive margins were more likely to develop a neck node recurrence (12%) than those with negative margins (10%). Again this difference was not significant (Table III).

In Figure 1 the proportion of patients developing a 'second' recurrence at the primary site is shown. This is the number of patients who develop a recurrence following excision of their post radiotherapy recurrence.

Figure 2 shows tumour-specific survival from the time of diagnosis of the primary site recurrence (second recurrence) to death or last follow up. The median time from tumour recurrence (after radiotherapy) to second recurrence (after surgery) was 1.1 years. Five year survival from the date of diagnosis of the primary tumour to the date of death or last follow-up was 41% for those patients with positive margins (95% CI ± 15.8%) and for those patients with negative margins was 48% (95% CI ± 6.9%). This difference in survival was not significant ($\chi^2_1 = 1.5333$, $P = 0.2163$). At 5 years from the diagnosis of the post radiotherapy recurrence the disease-free tumour-specific survival of patients with positive margins was 31% (95% CI ± 15.5%) compared with 43% for patients with negative margins (95% CI ± 6.8%). This difference in survival was significant ($\chi^2_1 = 5.308$, $P = 0.022$).

Survival data were further analysed for all patients for prognostic factors using Cox's proportional hazards model from the time of treatment of the post radiotherapy recurrence to the time of death or last follow-up. Site had a significant effect on survival (estimate 0.0154, $P = 0.0001$) with hypopharyngeal carcinoma associated with a poor prognosis and laryngeal carcinoma associated with a good prognosis. In addition T stage at recurrence was an important prognostic factor (estimate = −0.1778, $P = 0.0038$); patients with stage 3 or 4 disease at the primary

**Figure 1** Curve showing proportion of patients developing a 'second' recurrence at the primary site. □, positive margins (66% at 5 years); +, negative margins (47% at 5 years). Log-rank $\chi^2_1 = 4.7752$. $P = 0.0289$.

**Figure 2** Curve showing tumour-specific survival from the time of diagnosis of primary site recurrence to death or last follow-up. □, negative margins (5 year survival, 43%); +, positive margins (5 year survival, 31%). $\chi^2_1 = 5.308$. $P = 0.022$.

**Table III** 'Second' recurrence rates following surgery for post-radiotherapy recurrence (percentages in parenthesis), 1967–95, median follow-up 12.4 years (range 12 months–32 years)

| | *Negative margins* (n = *303*) | *Positive margins* (n = *49*) | $\chi^2_1$ P |
|---|---|---|---|
| Recurrence at primary site | 96 (32%) | 23 (47%) | $\chi^2_1$ = 3.7315<br>$P$ = 0.0534 |
| Recurrence in neck nodes | 29 (10%) | 6 (12%) | $\chi^2_1$ = 0.1044<br>$P$ = 0.7467 |

**Table IV** Details of patients dying of the original carcinoma, 1967–95, median follow-up 12.4 years (range 12 months–32 years) (percentages in parenthesis)

| *Details of death* | *Negative margins* (n = 127/303) (42%) | *Positive margins* (n = 26/49) (53%) | |
|---|---|---|---|
| Died with distant metastases and no local disease | 43 (14%) | 5 (10%) | |
| Died with disease at primary site | 63 (21%) | 15 (31%) | $\chi^2_2$ = 2.2720 |
| Died with disease in regional lymph nodes | 21 (7%) | 6 (12%) | *P* = 0.3211 |
| Total | 127 (42%) | 26 (53%) | |

site having a poor survival. Cox's regression from time of the diagnosis of the original tumour to the time of death or last follow-up was in addition significant for gender and neck node recurrence, with females having a better survival than males (estimate = 0.3709, $P = 0.0458$) and presence of neck nodes at recurrence also being associated with a poor survival (estimate = −0.0893, $P = 0.0187$). Of particular interest is that the presence or absence of a positive resection margin was not an independent predictor of survival (estimate = −0.2012, $P = 0.4944$).

In Table IV the mode of death for all patients dying of their recurrent tumour is detailed.

### Discussion

In the present study positive resection margins occurred in 14% of patients. The presence of a positive resection margin implies that the tumour was incompletely resected. In a previous study from this Unit (Cook *et al.*, 1993) we analysed resection margins in patients who had resection for their initial tumour and demonstrated a 7% difference in survival at 5 years. In the present study none of the patients received post-operative reirradiation. Sixty-six per cent of patients with a positive margin went on to develop a recurrence at the primary site compared with 47% in those patients with a negative margin. This significant difference is reflected in survival where the survival at 5 years in the group of patients with a positive resection margin was 12% less than for those with clear margins. The detrimental effect of positive resection margins has been noted in several previous studies (Lee, 1974; Byers *et al.*, 1978; Looser *et al.*, 1978; Chen *et al.*, 1987; Scholl *et al.*, 1986).

Other authors have noted that a positive margin is not necessarily disastrous, Byers *et al.* (1978) found that only 18% of patients having a hemilaryngectomy for carcinoma and who had a positive margin went on to develop a biopsy-proven local recurrence. In the series of Scholl *et al.* (1986) 54% of patients having resections for squamous carcinoma of the tongue had a positive margin on operative frozen section. The operation proceeded to remove the remaining tumour but it was found that the patients who had the positive frozen sections had a significantly increased risk of local recurrence and a decreased survival. Post-operative radiotherapy appeared to mitigate this effect. Vikram *et al.* (1984) studied 114 previously untreated patients with advanced resectable squamous carcinoma of the head and neck and found a local recurrence rate of 39% in patients with satisfactory margins compared with a recurrence rate of 73% for unsatisfactory margins. If post-operative radiotherapy was administered the recurrence rate at the primary site in patients with satisfactory margins fell to 2% and those with unsatisfactory margins fell to 10.5%. An unsatisfactory margin was defined as one that was microscopically involved with carcinoma, or where the carcinoma extended to within 5 mm of the resection margin.

The present study demonstrates that a positive margin was associated with oral cavity cancer but not with other sites. This finding is well known to head and neck surgical oncologists and it was noted by Harrison (1983) that wide excision margins were necessary for tongue cancer. This is presumably because muscle is a poor barrier to spread of tumour and in addition tumour can be 'milked' along the oral lymphatic system facilitating wide local spread.

Over a third of the patients with a positive resection margin were still alive and disease-free at 5 years with a high chance of being cured, and over half of these patients did not have a recurrence at the primary site. This finding is surprising but has been noted before (Byers *et al.*, 1978; Scholl *et al.*, 1986; Cook *et al.*, 1993).

This anomaly is fascinating and one can only postulate the reasons for it. Where excision was carried out with diathermy, this may have killed any remaining cancer cells within the tissue left behind. Scarring may also have rendered the remaining cancer cells non-viable. In addition the acute inflammatory reaction of the healing process may have killed cancer cells and it is always possible that the body's immune system was able to deal with the relatively low volume of cancer cells left following surgery. Also in a few patients the tumour is likely to have reached, but not crossed, the margin of resection.

Conversely, a negative resection margin does not guarantee that residual tumour is not present within the unresected tissue. Although the margins are always carefully studied, they form a three-dimensional structure and hence it is possible that tumour cells may be missed by the pathologist as a result of sampling errors during the preparation of the specimens. This may account for what may represent an erroneously high number of patients with allegedly negative margins who go on to develop a primary recurrence.

Whereas more than half the patients with positive resection margins do not go on to develop recurrent tumour at the primary site a high proportion do and this proportion is higher than those who have negative resection margins. Because of this our department's policy is now to consider further treatment if subsequent paraffin section histology demonstrates a positive resection margin. The best treatment is probably re-excision if this is possible. Reirradiation may be an option but if the patient is not suitable for either of these treatment modalities chemotherapy may be offered. There remains, of course, a group of patients who are unfit for further treatment and these patients still have a reasonable chance of cure of their recurrent cancer even if the positive resection margin is subjected to no further treatment.

The most satisfactory treatment is, of course, to completely excise the tumour at the first operation and per-operative frozen section histological examination is helpful in achieving this end.

**Acknowledgements**
The authors would like to acknowledge the work of Professor Philip Stell, who built up the University of Liverpool Head and Neck database over almost 30 years. We would also like to thank Mrs Brenda Cowley, who typed the manuscript, and Mrs Tracey Whittle, who drew the figures. The work was supported by a grant from The North West Cancer Research Fund.

### References

ARMITAGE P AND BERRY G. (1987). Survival analysis. In *Statistical Methods in Medical Research*, 2nd edn, Armitage P and Berry G (eds) pp. 421–439. Blackwell Scientific Publications: Oxford.

BOCCA E, PIGNATARO O AND MOSCIARO O. (1968). Supraglottic surgery of the larynx. *Ann. Otol. Rhinol. Laryngol.*, **77**, 1005–1026.

BYERS RM, BLAND KI, BORLASE B AND LUNA M. (1978). The prognostic and therapeutic value of frozen section determinations in the surgical treatment of squamous carcinoma of the head and neck. *Am. J. Surg.*, **136**, 525–528.

CHEN TY, EMRICH LJ AND DRISCOLL DL. (1987). The clinical significance of pathological findings in surgically resected margins of the primary tumor in head and neck carcinoma. *Int. J. Radiat. Oncol. Biol. Phys.*, **13**, 833–837.

COOK JA, JONES AS, PHILLIPS DE AND SOLER LLUCH E. (1993). Implications of tumour in resection margins following surgical treatment for squamous cell carcinoma of the head and neck. *Clin. Otol.*, **18**, 37–41.

COX D.R. (1972). Regression models and life-tables (with discussion). *J. R. Stat. Soc. B.*, **34**, 187–220.

HARRISON DFN. (1972). Role of surgery in the management of postcricoid and cervical esophageal neoplasms. *Ann. Otol. Rhinol. Laryngol.*, **81**, 465–468.

HARRISON DFN. (1983). The questionable value of total glossectomy. *Head Neck Surg.*, **6**, 632–638.

HERMANEK P AND SOBIN LH. (1992). *UICC TNM Classification of Malignant Tumours*, 4th edn, 2nd rev. Springer: Berlin.

LEE JG. (1974). Detection of residual carcinoma of the oral cavity, oropharynx, hypopharynx and larynx: a study of surgical margins. *Trans. Am. Acad. Ophthalmol. Otolaryngol.*, **78**, 49–53.

LOOSER KG, SHAH JP AND STRONG EW. (1978). The significance of 'positive' margins in surgically resected epidermoid carcinomas. *Head Neck Surg.*, **1**, 107–111.

MCCARTY PJ AND MILLION RR. (1994). History of diagnosis and treatment of cancer in the head and neck. In *Management of Head and Neck Cancer: a multidisciplinary approach*, 2nd edn. RR Million and NJ Cassisi (eds) pp. 1–29. JB Lippincott: Philadelphia.

PETO R, PIKE MC, ARMITAGE P, BRESLOW NE, COX DR, HOWARD SV, MANTEL N, MCPHERSON K, PETO J AND SMITH PG. (1977). Design and analysis of randomised clinical trials requiring prolonged observation of each subject. *Br. J. Cancer*, **35**, 1–39.

SAS INSTITUTE INC. (1985). *User's Guide: Statistics Version 5th edn.* SAS Institute: Cary, NC.

SCHOLL P, BYERS RM, BATSAKIS JG, WOLF P AND SANTINI M. (1986). Microscopic cut-through of cancer in the surgical treatment of squamous carcinoma of the tongue. Prognostic and therapeutic implications. *Am. J. Surg.*, **152**, 354–360.

VERONESI U. (1994). How important is the assessment of resection margins in conservative surgery for breast cancer? *Cancer*, **74**, 1660–1661.

VIKRAM B, STRONG EW, SHAH JP AND SPIRO R. (1984). Failure at the primary site following multimodality treatment in advanced head and neck cancer. *Head and Neck Surg.*, **6**, 720–723.

ZUBROD CG, SCHNEIDERMAN M, FREI E, BRINDLEY C, GOLD GL, SHNIDER B, OVIEDO R, GORMAN J, JONES R, JONSSON U, COLSKY J, CHALMERS T, FERGUSON B, DEDERICK M, HOLLAND J, SELAWRY O, REGELSON W, LASAGNA L AND OWENS AH. (1960). Appraisal of methods for the study of chemotherapy of cancer in man: comparative therapeutic trial of nitrogen mustard and triethylene thiophosphoramide. *J. Chron. Dis.*, **11**, 7–33.